# Melatonin Supplementation in Sex-Sorted Nili-Ravi Buffalo Semen: Effect on Sperm Quality, Subsequent in Vitro Embryo Development, and Pregnancy Outcomes

**DOI:** 10.3390/antiox15030344

**Published:** 2026-03-09

**Authors:** Xiaoxia Li, Danna Xu, Huiyan Xu, Pinghua Cao

**Affiliations:** 1 College of Animal Science and Technology, Henan University of Science and Technology, Luoyang 471023, China; xiaoxiali@haust.edu.cn; 2 Henan Provincial Key Laboratory for Grass-Feeding Animal, Henan University of Science and Technology, Luoyang 471023, China; 3 Luoyang Urban and Rural Integration Demonstration Zone Development Center of Agricultural and Forestry Technology, Luoyang 471935, China; lyybnb@163.com; 4 State Key Laboratory for Conservation and Utilization of Subtropical Agro-Bioresources, Guangxi High Education Key Laboratory for Animal Reproduction and Biotechnology, Guangxi University, Nanning 530004, China; hyxu@gxu.edu.cn

**Keywords:** buffalo, sex-sorted sperm, melatonin, sperm quality, embryo development, pregnancy outcomes

## Abstract

Melatonin (MLT) is a potent antioxidant that reduces oxidative stress (OS)-induced sperm damage. However, few studies have explored its effects in the field investigated here. This study aimed to evaluate the effects of MLT supplementation in extenders used for staining, sorting, and freezing on the quality of sorted Nili-Ravi buffalo sperm, embryo development after in vitro fertilization (IVF), and pregnancy outcomes following artificial insemination (AI). Computer-aided sperm analyzer (CASA) showed that progressive motility (PM) and velocity parameters of sorted, frozen–thawed sexed sperm were higher in the MLT-treated groups (*p* < 0.05). Fluorescence microscopy demonstrated that MLT significantly improved sperm plasma membrane integrity after staining and increased the proportion of frozen–thawed sex-sorted sperm with high mitochondrial membrane potential (HMMP) (*p* < 0.05). Raman spectroscopy further identified several distinctive Raman bands at 936, 1300, and 1651–1652 cm^−1^, which may serve as biomarkers for assessing sperm quality. Additionally, cleavage rate, blastocyst formation, and pregnancy rates following IVF and AI were higher in the MLT-treated group (*p* < 0.05). In conclusion, MLT can serve as a valuable additive during sperm sex-sorting procedures to enhance sperm quality, thereby improving embryo developmental competence and pregnancy outcomes.

## 1. Introduction

Buffalo (*Bubalus bubalis*) is a significant livestock species that provides meat, milk, and draught power, particularly in tropical and subtropical regions. With rising demand for high-quality dairy products, enhancing the genetic potential of river type buffalo (*Bubalus bubalis bubalis*), known for superior milk production compared to swamp buffalo (*Bubalus bubalis kerebau*), has become increasingly critical [1]. However, swamp buffaloes predominate in China, necessitating strategies such as crossbreeding with river buffalo to enhance local milk yields effectively [2]. Utilizing flow cytometry to sort X- and Y-bearing sperm in combination with AI [3] or IVF [4] can control offspring sex ratio and rapidly expand buffalo herds by increasing female calf production [1]. Additionally, globally, over 40% of embryo transfer operations involve IVF-derived embryos, with sex-sorted sperm significantly contributing to commercial sex-specific embryo production [5]. Nevertheless, sex-sorted bovine sperm typically shows compromised embryo developmental competence and conception rates during IVF and AI, though the exact mechanisms remain unclear [6].

Sex-sorting by flow cytometry and cryopreservation significantly increases reactive oxygen species (ROS) levels in sperm cells [7]. Excessive ROS in semen causes acrosome damage, DNA fragmentation, premature capacitation, reduced mitochondrial membrane potential, and decreased sperm motility, leading to poor fertilization and embryo development [7,8,9,10,11,12,13]. Furthermore, high sperm dilution during sorting and freezing reduces antioxidant levels [14,15]. Thus, strategies focused on scavenging excessive ROS by adding antioxidants to semen extenders are of significant interest [7,11,16,17,18].

Melatonin (MLT), a powerful antioxidant, can reduce ROS levels and prevent the depletion of endogenous antioxidant enzymes [19,20]. MLT (N-acetyl-5 methoxytryptamine), secreted by the pineal gland, has both lipophilic and hydrophilic properties [21]. Previous studies have shown that MLT directly improves sperm membrane and DNA integrity [22,23,24], reduces membrane lipid peroxidation (LPO) [23,25,26], preserves mitochondrial function [25,27], and maintains cellular homeostasis against OS [28]. Consequently, antioxidants improve sperm viability, increasing embryo developmental potential [13,27,29,30] and pregnancy rates following AI [12,31]. Our previous reports indicated that MLT protects Nili-Ravi buffalo sperm during sex-sorting procedures, with an optimal concentration of 10^−4^ M [8]. However, no research has assessed the effects of MLT on sperm motility parameters, the biochemical composition of the sperm midpiece during the sperm sex-sorting process in Nili-Ravi buffalo, embryo development following IVF, or pregnancy rates following AI.

Thus, this study evaluated the protective effects of supplementing staining, sorting, and freezing media with 10^−4^ M MLT during sperm sex-sorting and cryopreservation processes. Parameters analyzed included sperm motility, mitochondrial activity, plasma membrane integrity, and apoptosis in buffalo sperm. Furthermore, morphological and kinematic traits were assessed, and biochemical alterations within the sperm midpiece, induced by treatment with or without MLT, were examined using Raman spectroscopy. The fertilizing capacity of frozen–thawed sex-sorted sperm, with and without MLT supplementation, was also determined through IVF and AI trials. These results aim to optimize sex-sorting protocols and promote broader commercial utilization of sex-sorted buffalo sperm.

## 2. Materials and Methods

Unless otherwise stated, all chemicals were purchased from Sigma Chemical Company (St. Louis, MO, USA).

### 2.1. Animals

Semen was collected from ten proven fertile Nili-Ravi buffalo bulls (river-type) using an artificial vagina at Guangxi Livestock and Poultry Breeding Station. Swamp-type buffalo cows and F1 × swamp crossbred cows used for AI were raised on household farms located in Wuming District, Nanning, China.

### 2.2. Semen Collection and Sperm Preparation

The semen collected met the quality control standards of ≥6 × 10^8^ sperm/mL concentration and >65% PM. Collected semen was diluted with staining extender to achieve a concentration of 1 × 10^8^ sperm/mL. In the treatment group, 10 μL of 0.02 M MLT (M5250, Sigma) was added to 2 mL staining extender to achieve a final concentration of 10^−4^ M [8]. The control group did not receive MLT supplementation. Diluted sperm was stained with 40 μg/mL Hoechst 33342 solution at 34 °C for 45 min. Post-incubation, samples were marked with food dye solution (FD&C#40, 0.01305%) to identify nonviable sperm, and then filtered through a 50 µm cell strainer. Stained sperm samples were divided into two parts: one was diluted to 1 × 10^7^ sperm/mL with Dulbecco’s Phosphate-Buffered Saline (DPBS) for subsequent analysis, and the other was reserved for sperm sorting.

Flow cytometer sorting utilized a Dako MoFlo^®^ SX cytometer (DakoCytomation, Fort Collins, CO, USA) equipped with an argon laser. Sorted sperm was collected into 50-mL tubes containing 2 mL of 20% (*v*/*v*) Tris-egg yolk extender, supplemented with (treatment) or without melatonin (control). After collecting approximately 10 million sperm per tube, the samples were split into two fractions, one resuspended in DPBS at 37 °C, and the other chilled at 4 °C for 60 min. The chilled sorted semen underwent centrifugation, and the resulting pellets were resuspended in Tris-based freezing extender, again with (treatment) or without MLT supplementation (control), before freezing and storage in liquid nitrogen. Frozen–thawed sperm was resuspended in DPBS for analysis and subsequent IVF and AI procedures.

### 2.3. Sperm Quality Assay

#### 2.3.1. Assessment of Sperm Motility by Computer-Aided Sperm Analyzer (CASA)

Sperm motility parameters were assessed using a CASA system. Eight random fields were selected at 38 °C for each processed sample, and at least 80 sperm cells per field were analyzed. Motility parameters included: total motility (TM%), PM%, average path velocity (VAP, μm/s), straight-line velocity (VSL, μm/s), curvilinear velocity (VCL, μm/s), beat cross frequency (BCF, Hz), and linear coefficient (LIN%, VSL/VCL). Stained and sorted semen samples were incubated at 38 °C for 2 min before CASA analyses. Frozen semen samples were thawed at 38 °C for 30 s in water. Then, 10 μL from stained, sorted, and frozen–thawed samples were analyzed separately using CASA. Semen samples from ten Nili-Ravi buffalo bulls were individually analyzed without pooling, and each bull served as an independent biological replicate.

#### 2.3.2. Analysis of Sperm Mitochondrial Activity, Apoptosis and Plasma Membrane Integrity by Fluorescence Microscope

Semen samples underwent microscopic analysis using a Nikon (Tokyo, Japan) phase-contrast microscope equipped with a digital imaging. Mitochondrial activity was evaluated using a mitochondrial membrane potential (MMP) assay (JC-1, Beyotime, Shanghai, China), apoptosis detection employed an Annexin-V/propidium iodide (PI) kit (BD Pharmingen, Franklin Lakes, NJ, USA), and plasma membrane integrity assessments utilized SYBR-14 combined with PI staining (L-7011 LIVE/DEAD^®^ Sperm Viability Kit, Thermo Fisher Scientific, Eugene, OR, USA).

The JC-1 assay followed manufacturer instructions: sperm cells incubated with JC-1 staining solution (5 μg/mL) at 37 °C for 30 min in darkness were resuspended in JC-1 buffer for microscopy (400×). JC-1 monomers emit green fluorescence [excitation wavelengths (Ex)/emission wavelengths (Em): 510/530 nm], whereas J-aggregates emit red–orange fluorescence (Ex/Em: 585/590 nm). Red–orange fluorescence indicated HMMP, whereas green fluorescence signaled low mitochondrial membrane potential (LMMP) [32].

Annexin-V/PI staining, slightly modified, detected phosphatidylserine externalization indicative of apoptosis. Semen samples suspended in Annexin-binding buffer (1 × 10^7^ sperm/mL) were mixed with Annexin V-FITC (5 μL) conjugate and PI (5 μL), incubated in darkness for 15 min at room temperature, and diluted further with binding buffer. Fluorescence microscopy analyzed samples within 5 min, where apoptotic sperm fluoresced green and necrotic cells fluoresced red [33].

SYBR-14/PI staining assessed plasma membrane integrity. Semen samples suspended in HEPES buffer (1 × 10^7^ sperm/mL) received SYBR-14 and PI dyes, incubated for 5 min in darkness at 37 °C. Slides were prepared, and at least 200 sperm per replicate across ten replicates were examined microscopically. Viable sperm exhibited bright green fluorescence, while nonviable sperm presented red fluorescence, indicating compromised membranes [34].

#### 2.3.3. Raman Spectra Analysis

Laser Tweezers Raman Spectroscopy (LTRS, Princeton Instruments, Trenton, NJ, USA) was utilized to assess biochemical changes within the sperm midpiece region. Samples were prepared using various extenders, either supplemented with or without MLT during the sorting and cryopreservation processes. The LTRS setup incorporated a 785 nm laser transmitted into an inverted Nikon TE2000 microscope using a 100 × objective lens (numerical aperture 1.30) to create an optical trap. Laser power averaged approximately 15 mW at the objective input, and the spectral resolution was maintained at 6 cm^−1^. Sperm samples were initially resuspended in DPBS at ambient temperature, and Raman spectra were collected for 60 sperm midpiece (30 sperm/group) at each procedural stage (stained, sorted, frozen–thawed), employing an integration period of 120 s per cell. Spectra were obtained within the range of 800–1800 cm^−1^ under darkroom conditions, with background spectra recorded from DPBS controls devoid of sperm under identical settings.

The dark signal background was subtracted from each spectrum, and spectra were smoothed using a Savitsky–Golay filter. Baseline correction for all sperm spectra was performed using Fityk 0.9.8 software [35]. Averaged Raman spectra from all sperm samples were plotted using Origin 2024 software (OriginLab Corporation, Northampton, MA, USA). Principal component analysis (PCA), a multivariate statistical analysis method, was employed to develop effective diagnostic algorithms for discriminating between control and MLT-treated buffalo sperm samples.

### 2.4. In Vitro Embryo Production

#### 2.4.1. Oocyte Collection and In Vitro Maturation

Swamp buffalo ovaries were sourced from a local slaughterhouse in Nanning, China, and delivered to the laboratory within 6 h post-slaughter in sterile saline solution (0.9% NaCl) and maintained at 35 °C. Ovaries underwent trimming to eliminate surrounding tissue and were subsequently rinsed again with sterile saline. Cumulus-oocyte complexes (COCs) were aspirated from ovarian follicles (2–8 mm) using an 18-gauge needle coupled with a disposable 10 mL syringe. Retrieved COCs possessing more than three compact cumulus cell layers were thoroughly washed three times with maturation medium (TCM-199). Groups of approximately 30 COCs were then cultured in 50 µL droplets of maturation medium covered with mineral oil at 39 °C in a 5% CO_2_ humidified incubator for 22–24 h.

#### 2.4.2. IVF

Frozen–thawed semen samples categorized as non-sexed (NS), sexed for X (SX) and SX semen supplemented with MLT (SXM) were employed in IVF experiments. Straws containing semen were thawed in a 38 °C water bath for 10 s before being transferred into the bottom of conical tubes containing 3 mL PBS supplemented with 0.1% BSA. Following centrifugation, the supernatant was discarded, leaving a sperm pellet volume of 100 µL. The pellet was washed again with modified TALP (fertilization medium), recentrifuged, and finally resuspended in 100 µL of fresh TALP medium for IVF use. Semen samples from the same ejaculate of each of five Nili-Ravi buffalo bulls were used for IVF. The experiment was performed with ten replicates, with one straw from each bull included in each replicate.

Roughly 30 oocytes per treatment per replicate were washed twice in modified TALP medium and placed into fertilization droplets (50 µL) under mineral oil. Sperm was introduced at a concentration of 1 × 10^6^/mL, and co-incubated with oocytes for 6–8 h at 39 °C under conditions of 5% CO_2_ and complete humidity. The fertilization day was defined as Day 0.

#### 2.4.3. Embryo Culture

Following fertilization, presumptive zygotes were separated from the fertilization media, and cumulus cells were mechanically removed with a holding pipette. Zygotes were rinsed twice in culture medium (TCM199), transferred to 20 µL droplets containing 10% fetal calf serum (FCS), and co-cultured alongside buffalo cumulus cells at 39 °C, 5% CO_2_, and full humidity for a period of 9 d. Cleavage rates were assessed on Day 2, and blastocyst formation was recorded between Days 7–8. The culture medium was refreshed by replacing half the volume every two days.

### 2.5. Artificial Insemination

For AI procedures, 110 buffalo cows were randomly allocated into two groups and inseminated with sexed semen either with or without MLT after synchronized estrus. All cows, primarily pasture-raised, were inseminated by a single technician. Sexed semen samples with or without MLT were thawed immediately prior to deposition into the deep uterine horn corresponding to the ovulatory follicle. Pregnancy was confirmed via transrectal palpation three months post-insemination, with failure to reach full-term development classified as embryo loss or abortion.

### 2.6. Statistical Analysis

Statistical analyses were performed using SPSS 22.0 software (IBM Corporation, Armonk, NY, USA). The normality of data distribution was evaluated using the Shapiro–Wilk test, and homogeneity of variance was examined using Levene’s test. For data satisfying both assumptions, parametric tests were employed. CASA (control and MLT groups), fluorescence microscopy, and IVF data were analyzed using Student’s *t*-test. One-way ANOVA was conducted to identify differences in CASA parameters among the three groups (stained, sorted, and frozen–thawed stages), followed by Tukey’s multiple range test for pairwise comparisons. AI outcomes were evaluated using the chi-square test. Embryo data were analyzed by cleavage and blastocyst rates based on total inseminated oocytes. Raman spectroscopy data analysis employed Origin 2024 software and Unscramb software (CAMO, Oslo, Norway). Data were presented as means ± SD, and statistical significance was set at *p* < 0.05.

## 3. Results

### 3.1. Sperm Quality Analyses

Table 1 presents a summary of the motility parameters assessed in supplementation semen extenders with 10^−4^ M MLT during the sperm sex sorting process. Compared to control groups, sorted and frozen–thawed sperm samples treated with MLT exhibited significantly improved (*p* < 0.05) TM%, PM%, VAP, VSL and VCL, whereas stained samples showed no notable differences. Conversely, BCF and LIN parameters demonstrated no significant variation (*p* > 0.05) between control and MLT-treated samples across all processing stages. Moreover, regardless of MLT supplementation, the motility parameters TM%, PM%, VAP, VSL, VCL, BCF, and LIN were significantly lower (*p* < 0.05) in frozen–thawed sex-sorted sperm compared with stained sperm.

The influence of 10^−4^ M MLT supplementation on mitochondrial activity, apoptosis rates, and plasma membrane integrity throughout the sorting process was assessed via fluorescence microscopy, as detailed in Table 2. At all evaluated stages, apoptotic sperm proportions showed no significant differences between the control and MLT-treated groups (*p* > 0.05). Similarly, the proportion of sperm exhibiting red–orange fluorescence (JC-1 staining) remained unaffected during staining and sorting phases (*p* > 0.05). Nonetheless, frozen–thawed samples supplemented with MLT demonstrated a significantly elevated percentage of sperm with HMMP, compared to the control group (65.85% vs. 60.19%, *p* < 0.05). Plasma membrane integrity, as determined by SYBR-14/PI staining, did not vary significantly between sorted and frozen–thawed samples, regardless of MLT supplementation (*p* > 0.05). However, stained samples treated with MLT exhibited a higher proportion of sperm with intact membranes than in the control group (76.26% vs. 70.24%, *p* < 0.05).

**Figure 1:**
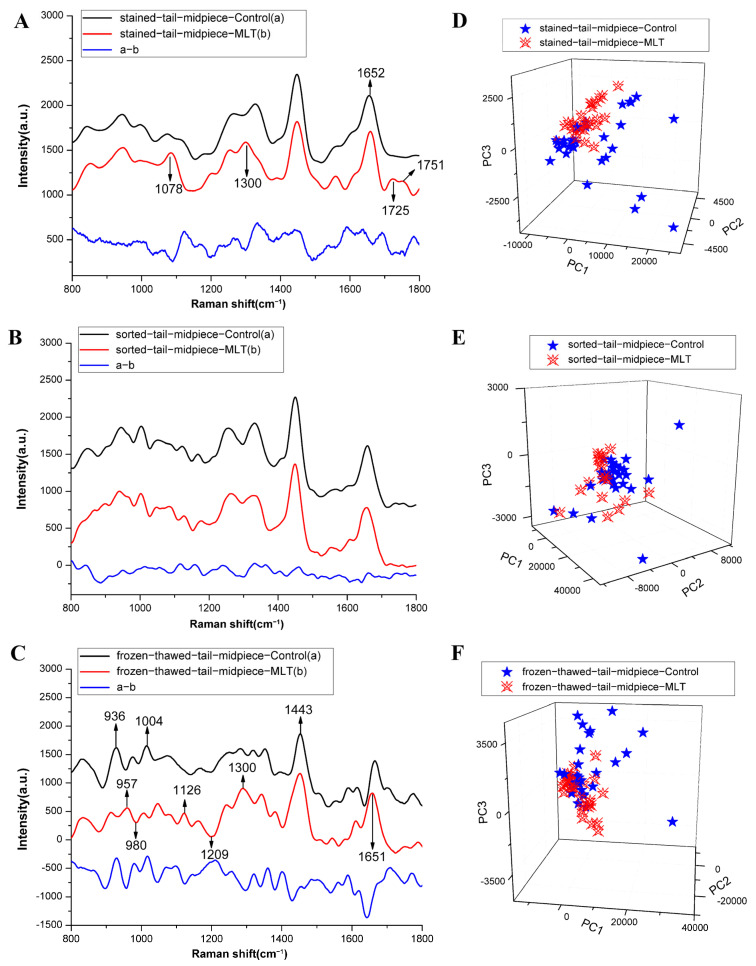
Average and difference Raman spectra of the sperm tail midpiece in in stained (**A**), sorted (**B**) and frozen–thawed (**C**) samples with (b) or without (a) MLT treatment. Three-dimensional PCA scatter plots illustrate separation between treated and control groups during the stained (**D**), sorted (**E**) and frozen–thawed (**F**) phases.

Overall, these results revealed that mitochondrial activity was the only parameter significantly affected by MLT supplementation during the frozen–thawed stage. Consequently, biochemical changes in the sperm midpiece (characterized by the presence of mitochondria) region during sorting and freezing processes were further explored via LTRS. Figure 1 illustrates the average and differential Raman spectra of the sperm midpiece from samples treated with or without MLT at each processing stage. Notable spectral changes in distinct Raman peaks between treated and untreated groups emerged primarily during staining and freezing stages, with peak assignments outlined in Table 3 [9,36,37,38,39,40,41,42].

**Figure 2:**
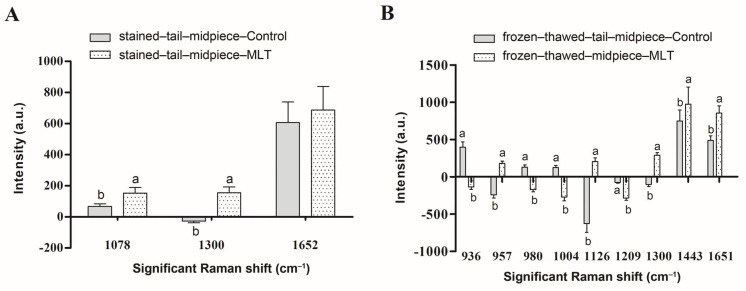
Histogram exhibited changes of the intensities in the most significant characteristic peaks across defined spectral regions. Histogram (**A**) corresponds to the stained phase of flow-sorting, displaying data for three Raman spectral regions in the control group (grey) and the melatonin (MLT)-treated group (punctuated). Histogram (**B**) corresponds to the frozen–thawed phase of flow-sorting, displaying data for nine Raman spectral regions in two groups. Distinct letters (a, b) indicate significant variations (*p* < 0.05) between control and MLT treatment.

During staining (Figure 1A and Figure 2A), the intensities of the characteristic peaks at 1078 cm^−1^ (C–C or C–O stretch of lipid) and 1300 cm^−1^ (CH_2_ deformation of lipid), which were assigned to the lipids, significantly increased in the samples with MLT. No marked spectral differences were found during sorting (Figure 1B). In contrast, frozen–thawed samples (Figure 1C) displayed substantial changes in nine Raman peaks following MLT supplementation, five increased and four decreased (Figure 2B). The peaks showing reduced intensities included 936 cm^−1^ (glycogen vibration), 1004 cm^−1^ (phenylalanine symmetric ring breathing), 1209 cm^−1^ (tryptophan and phenylalanine vibration), and 980 cm^−1^ (O–P–O^−^ stretching of nucleic acids). Enhanced peaks encompassed 957 cm^−1^ (cholesterol vibration), 1126 cm^−1^ (C–C stretching of lipids), 1300 and 1443 cm^−1^ (CH_2_ deformation of lipids), and 1651 cm^−1^ (lipid vibration).

PCA results, depicted as three-dimensional scatter plots based on the first three principal components (PC1–PC3), indicated that approximately 92.9%, 91.1%, and 92.7% of the variance was explained during stained, sorted, and frozen–thawed stages, respectively (Figure 1D–F). Notably, samples treated with MLT during staining and freezing phases showed tighter clustering patterns, suggesting greater biochemical homogeneity.

### 3.2. IVF and AI Outcomes Analyses

IVF results demonstrated that MLT-treated sex-sorted sperm yielded significantly improved cleavage (53.74% vs. 41.51%) and blastocyst formation rates (27.21% vs. 18.24%) compared to untreated counterparts (*p* < 0.05) (Table 4). Furthermore, the control non-sexed sperm resulted in significantly higher (*p* < 0.05) cleavage (55.66% vs. 41.51%) and blastocyst rates (28.30% vs. 18.24%) than sexed sperm without melatonin treatment, but no significant difference (*p* > 0.05) compared to sexed sperm with melatonin.

To further assess the efficacy of MLT supplementation, 110 buffalo cows synchronized for estrus were inseminated with frozen–thawed sex-sorted sperm either supplemented (*n* = 65) or unsupplemented (*n* = 45) with MLT into the deep uterine horns. Pregnancy outcomes assessed three months post-insemination indicated a significantly increased pregnancy rate in cows inseminated with MLT-treated sperm relative to the control group (76.92% vs. 51.11%, *p* < 0.05) (Table 5). Nonetheless, there were no significant variations between groups concerning the proportion of female calves born (*p* > 0.05) (Table 5).

## 4. Discussion

Sex-sorted sperm exhibit reduced efficacy in vitro and in vivo due to detrimental effects from the sorting procedure on sperm cells [43]. This occurs because sperm are sensitive to multiple factors during flow cytometry sorting, including high dilution, staining, elevated pressure, laser exposure, freezing, thawing, centrifugation, and changes in media composition [44,45]. These factors may shorten sperm lifespan [46] and reduce embryo developmental potential [47], primarily through excessive ROS generation during flow cytometry. Thus, decreasing ROS levels in sex-sorted sperm remains critical. In this study, different antioxidants were added during sorting and freezing processes to protect sexed sperm quality against ROS-induced LPO [11,18], thereby reducing OS.

Many studies reported that MLT, a potent antioxidant, protects sperm quality from oxidative damage and enhances sperm parameters in boars [48], red deer [49], stallions [50], bulls [51], roosters [52], rabbits [53], and rams [54]. Currently, the influence of MLT supplementation on the motility parameters of sex-sorted buffalo sperm, subsequent embryo development in vitro, and resulting pregnancy outcomes is not thoroughly investigated. Thus, this research aimed to examine whether MLT effectively mitigates sperm quality deterioration associated with flow cytometry sorting and cryopreservation processes.

CASA provides more detailed and objective quantification of sperm motion characteristics compared to visual assessment in buffalo bulls [55,56]. The sperm motion pattern indirectly reflects biochemical and physical conditions affecting spermatozoa [57]. In this study, motion parameters (TM%, PM%, VAP, VSL and VCL) of sexed buffalo sperm were higher with MLT supplementation compared to controls in sorted and frozen–thawed stages. These findings align with previous studies showing increased TM%, PM%, VAP, VCL, BCF, and LIN in fresh and frozen–thawed sperm after treatment with slow-release MLT implants in mithun (*Bos frontalis*) [22]. Ashrafi et al. has also reported that adding 0.1 mM MLT to freezing extender improved TM% and PM% in bull sperm, but had no effect on BCF and LIN [23]. Consistently, our findings indicated no significant variations in BCF and LIN among stained, sorted, and frozen–thawed buffalo sperm irrespective of MLT treatment. Parameters of sperm motility and velocity serve as indirect indicators of mitochondrial performance and energy availability [58], factors closely correlated with sperm fertility potential [59]. MLT supplementation significantly enhanced TM%, PM%, VAP, and VSL in buffalo sexed sperm during sorted and frozen–thawed stages, while VCL improved only in the frozen–thawed stage. High VCL indicates elevated sperm energy status, which is crucial for successful fertilization [60]. Although ROS produced during the sorting and freezing–thawing processes decreases motility [45], MLT improves sperm motility by stimulating the cellular Ca^2+^ influx [61], scavenging intracellular free radicals, and inhibiting membrane LPO [22,23,62,63,64]. Thus, MLT supplementation during the sorting and freezing processes improves sperm motility and velocity parameters.

MLT and its metabolites are well-known antioxidants and potent free radical scavengers [65]. MLT protects mitochondria and plasma membranes from free radicals, lipid peroxides, and ROS, resulting in increased MMP, cell viability, membrane fluidity, and reduced LPO [66]. In this study, mitochondrial activity in frozen–thawed sexed buffalo sperm with MLT supplementation was significantly higher than in controls. These findings agree with Pang et al., who reported increased sperm mitochondrial activity following 10^−5^ and 10^−3^ M MLT supplementation in bovine semen freezing extenders [27]. Our previous research demonstrated that supplementation with 10^−4^ M MLT significantly enhanced ATP production from glucose and mitochondrial activity in frozen–thawed sex-sorted buffalo sperm [8]. Enhanced ATP production by MLT is likely attributable to increased activity within mitochondrial respiratory complexes [67,68]. Furthermore, MLT’s interaction with lipid bilayers promotes stabilization of mitochondrial inner membranes, thereby supporting sperm motility [69]. Post-sorting and freeze–thaw assessments revealed no considerable differences in sperm plasma membrane integrity and apoptotic rates between MLT-treated and untreated groups. Hence, sperm oxidative damage likely occurs predominantly during freezing–thawing rather than the staining or sorting phases. Collectively, MLT improved mitochondrial performance and motility without adversely impacting sperm viability during the freezing–thawing stage.

Raman spectroscopy, recognized as a rapid, non-invasive, and label-free analytical technique, facilitates real-time monitoring of subtle biochemical changes within biological samples through spectral shifts [39]. Several steps in sperm sex sorting induce ROS production and LPO; however, supplementation with antioxidants can effectively reduce oxidative damage [7,18,30,70]. Raman spectroscopy in this study revealed distinct spectral features in the 800–1800 cm^−1^ region for metabolically active sperm tail midpiece (mitochondria-rich) with or without MLT supplementation in staining and freezing extenders during sex sorting. After staining in medium with MLT, bands at 1078 and 1300 cm^−1^ (assigned to lipids) showed significantly greater intensities (Figure 1A and Figure 2A), indicating that MLT effectively maintains sperm membrane integrity [8,12]. Li et al. [30] demonstrated MLT decreases ROS in sex-sorted bull sperm, mitigating LPO damage [71]. However, no MLT-induced spectral differences appeared in sorted buffalo sperm tail midpiece (Figure 1B), possibly because sorting selects sperm with intact membranes [72]. This observation was consistent with fluorescence microscopy results showing no significant mitochondrial activity differences between MLT-treated and control groups. Finally, frozen-thawed sperm midpiece supplemented with MLT showed increased Raman intensities at 957 cm^−1^ (cholesterol) and at lipid-associated bands (1126, 1300, 1443, 1651 cm^−1^), whereas intensities decreased at 936 cm^−1^ (glycogen), 980 cm^−1^ (O–P–O^−^ stretch), 1004 cm^−1^ (Symmetric ring breathing mode of phenylalanine), 1209 cm^−1^ (Tryptophan and phenylalanine v mode). These results indicated MLT improved DNA and proteins stability, promoted efficient glycogen metabolism for ATP synthesis, and reduced membrane damage [8]. Monllor et al. [73] reported that preincubation with MLT enhances DNA compaction in human sperm and reduces DNA fragmentation. Cryopreservation-induced freeze–thaw cycles damage sperm, impairing motility, MMP, and intracellular ATP [74]. Previous reports showed MLT maintained higher ATP levels during sperm storage and improved fertilizing capacity after 72 h storage [48,75]. In this study, MLT-treated sperm showed decreased glycogen levels after freezing–thawing, indicating enhanced energy utilization to sustain superior motility. CASA analysis supported this, showing significantly increased TM% and PM% in MLT-supplemented sex-sorted buffalo sperm. MLT also improved mitochondrial activity, membrane integrity, and fertilization ability in bull sperm by reducing oxidative damage [30].

PCA, a multivariate statistical technique, systematically analyzed the Raman spectral data from buffalo sperm midpiece with and without MLT. PCA extracts key spectroscopic information and captures differences between treated and control groups [76]. Control buffalo sperm displayed broader distributions than MLT-treated sperm in staining and freezing–thawed stages. These results demonstrated MLT preserves sperm quality by maintaining structural integrity and biochemical uniformity during flow cytometric sorting [8]. Overall, our study identified distinct Raman bands at 936, 1300, and 1651–1652 cm^−1^ as potential biomarkers for evaluating sperm quality.

Excessive OS due to elevated ROS levels during sex-sorting processes can impair sperm function, negatively affecting fertilization and embryo development in vitro [77]. MLT helps protect sperm against ROS-induced damage during sperm sorting, a common technique in IVF and AI [8]. In this study, buffalo oocytes fertilized using SXM sperm demonstrated significantly superior cleavage and blastocyst formation rates compared to those fertilized by SX sperm alone. These results align with findings by Li et al., who similarly reported elevated cleavage and blastocyst development rates in bovine embryos produced in vitro using sex-sorted sperm treated with 10^−5^ M MLT [30]. MLT accelerated the first embryonic division and improved cleavage rates [78,79]. Perumal et al. also reported that MLT-treated bull sperm had a higher number of sperm bound to the zona pellucida of in vitro matured ova, suggesting improved in vitro fertilizing capability [22]. These outcomes match our findings that MLT supplementation enhanced buffalo embryo cleavage rates. Likewise, adding MLT during bovine sperm preparation improved fertilization efficiency and subsequent embryonic competence in vitro [13]. MLT-treated sperm might exhibit increased longevity, enhanced oocyte penetration, or beneficial changes in capacitation, resulting in improved fertilizing capability [22]. Additionally, our experiments demonstrated that MLT-driven improvement in sorted sperm parameters ultimately increased embryo cleavage and blastocyst rates, matching the developmental potential of buffalo oocytes fertilized with unsorted sperm. This result indicated that MLT helps optimize buffalo sperm sex-sorting procedure [8], enhances fertilization, and promotes subsequent in vitro embryonic development. Therefore, MLT’s antioxidant effect likely reduces OS during sorting and freezing–thawing processes, improving the quality of resulting buffalo embryos.

Combining flow cytometric sperm sorting with AI effectively modulates offspring sex ratios in mammals, accelerating herd expansion by selectively enriching female progeny [3]. Understanding basic sperm and semen characteristics is essential for developing effective AI and sperm sexing methods [38]. MLT supplementation during semen cryopreservation reduces OS-induced sperm damage, thereby improving sperm quality and fertilizing ability, contributing to higher pregnancy rates after AI [12]. Previous studies reported significantly increased AI pregnancy rates and enhanced farm animal productivity with MLT-supplemented semen [31,80]. Our study found that MLT supplementation during sex-sorting significantly increased the pregnancy rate, although it had no significant effect on the percentage of female offspring. These findings offer a promising approach to improve fertilization outcomes with sex-sorted buffalo sperm.

## 5. Conclusions

MLT supplementation during staining, sorting, and freezing processes significantly protected sperm quality, resulting in improved embryo development after IVF and enhanced pregnancy outcomes following AI in buffalo. This study provides a practicable reference for improving reproductive efficiency using sex-sorted buffalo sperm. However, given MLT’s role as a potent antioxidant, future research should focus on OS profiles (e.g., MDA, SOD, ROS levels) and related gene expression to elucidate regulatory mechanisms underlying MLT-mediated sperm protection during sex-sorting.

## Figures and Tables

**Table 1:** Motility parameter comparisons measured by CASA in buffalo sperm subjected to staining, sorting, and cryopreservation with or without MLT supplementation.

CASA Parameters	Groups	Stained	Sorted	Frozen–Thawed
TM (%)	Control	96.55 ± 2.28 ^A^	62.80 ± 7.89 ^bB^	58.88 ± 3.39 ^bB^
	MLT	96.71 ± 2.03 ^A^	76.87 ± 12.83 ^aB^	68.67 ± 9.90 ^aB^
PM (%)	Control	86.36 ± 2.37 ^A^	40.77 ± 6.44 ^bB^	32.37 ± 1.73 ^bC^
	MLT	87.57 ± 2.04 ^A^	54.23 ± 10.81 ^aB^	46.42 ± 4.45 ^aC^
VAP (μm/s)	Control	123.50 ± 10.09 ^A^	124.47 ± 11.67 ^bA^	80.53 ± 8.07 ^bB^
	MLT	120.96 ± 10.03 ^B^	138.57 ± 9.43 ^aA^	87.43 ± 6.07 ^aC^
VSL (μm/s)	Control	105.27 ± 12.35 ^A^	89.73 ± 10.02 ^bB^	55.67 ± 14.79 ^bC^
	MLT	105.48 ± 12.52 ^A^	97.17 ± 5.00 ^aA^	69.97 ± 9.35 ^aB^
VCL (μm/s)	Control	183.54 ± 26.09 ^B^	213.77 ± 24.61 ^bA^	126.30 ± 8.35 ^bC^
	MLT	174.36 ± 21.94 ^B^	254.96 ± 20.39 ^aA^	139.72 ± 7.64 ^aC^
BCF (Hz)	Control	34.16 ± 2.42 ^A^	29.19 ± 4.22 ^A^	14.72 ± 7.79 ^B^
	MLT	35.66 ± 3.18 ^A^	28.84 ± 2.06 ^B^	16.68 ± 4.89 ^C^
LIN (%)	Control	56.69 ± 6.87 ^A^	43.38 ± 7.05 ^B^	47.68 ± 8.92 ^B^
	MLT	59.70 ± 4.22 ^A^	40.40 ± 5.73 ^C^	53.75 ± 6.41 ^B^

Distinct letters (a, b) within columns represent significant differences (*p* < 0.05) between MLT-treated and control groups. Distinct letters (A, B, C) within rows indicate significant variations (*p* < 0.05) among sorting stages. *n* = 10 samples per group.

**Table 2:** Effect of melatonin on sperm apoptosis, HMMP and plasma membrane integrity in buffalo during sperm sex sorting procedures.

	Stained		Sorted		Frozen–Thawed	
	Control	MLT	Control	MLT	Control	MLT
Annexin V/PI(A+)	13.99 ± 1.48	12.48 ± 1.82	14.60 ± 3.43	11.97 ± 2.24	22.73 ± 6.26	17.31 ± 5.77
JC-1(HMMP)	66.28 ± 11.28	76.33 ± 12.76	78.21 ± 8.41	80.54 ± 11.33	60.19 ± 2.29 ^b^	65.85 ± 1.51 ^a^
SYBR-14/PI(SYBR+)	70.24 ± 3.68 ^b^	76.26 ± 5.02 ^a^	74.19 ± 4.17	76.21 ± 3.23	60.15 ± 2.94	62.25 ± 2.37

Distinct letters (a, b) within rows indicate significant variations (*p* < 0.05) between MLT-treated and control groups. *n* = 10 samples per group.

**Table 3:** Sperm Raman bands and their tentative assignments.

Band (cm^−1^)	Assignation
936	Glycogen
957	Cholesterol
980	O–P–O^−^ stretch (nucleic acids)
1004	Symmetric ring breathing mode of phenylalanine
1078	C–C or C–O stretch (lipid)
1126	C–C stretching mode of lipids
1209	Tryptophan and phenylalanine v mode
1300	CH_2_ deformation (lipid)
1443	CH_2_ deformation (lipid)
1651–1652	lipid
1725–1751	C=O stretch (lipid)

**Table 4:** Cleavage and blastocyst development formation rates after IVF using frozen–thawed non-sexed (NS), sexed for X (SX), and SX semen supplemented with MLT (SXM) buffalo semen.

Sperm	No. of Oocytes	Cleavage (%)	Blastocyst (%)
NS	424	55.66 ± 6.92 ^a^	28.30 ± 4.95 ^a^
SX	318	41.51 ± 8.61 ^b^	18.24 ± 3.58 ^b^
SXM	294	53.74 ± 11.65 ^a^	27.21 ± 4.40 ^a^

Distinct letters within columns indicate significant variations (*p* < 0.05). *n* = 10 samples per group.

**Table 5:** Pregnancy and calving rates following AI with frozen–thawed sex-sorted sperm, with melatonin (MLT) or without melatonin (Control).

Variable	No. of Inseminations	Pregnancy (%)	Calving		
			Total	Females	Percentage of Females (%)
Control	45	51.11% (23/45) ^b^	22	19	86.36%
MLT	65	76.92% (50/65) ^a^	49	44	89.80%

Distinct letters within columns indicate significant variations (*p* < 0.05).

## Data Availability

The original contributions presented in this study are included in the article. Further inquiries can be directed to the corresponding authors.
